# Was kosten eine OP-Minute sowie ein Tag auf der Intensiv- und Peripherstation? Modell zur Personalkostenkalkulation von Ärztlichem Dienst und Pflegedienst in einer universitären Unfallchirurgie

**DOI:** 10.1007/s00113-025-01644-0

**Published:** 2025-10-21

**Authors:** Katja Hierl, Laura Schörner, Volker Alt

**Affiliations:** 1 Klinik und Poliklinik für Unfallchirurgie, Universitätsklinikum Regensburg, Franz-Josef-Strauss-Allee 11, 93053 Regensburg, Deutschland; 2 Stabsstelle Zentralcontrolling, Personalcontrolling, Universitätsklinikum Regensburg , Franz-Josef-Strauß-Allee 11, 93053 Regensburg, Deutschland

**Keywords:** Personalaufwand, Krankenhaus, Ärzte, Pflege, Kosteneffizienz, Personnel expenses, Hospital, Medical service, Nursing service, Cost efficiency

## Abstract

**Hintergrund:**

Der Großteil der Gesamtkosten in den deutschen Krankenhäusern entfällt mit rund zwei Dritteln auf die Personalkosten für den Ärztlichen Dienst und Pflegedienst. Ziel dieser Arbeit war, eine modellhafte Kalkulation der Personalkosten von ärztlichem und pflegerischem Dienst für die kostenintensiven Bereiche OP, Intensivstation und Peripherstation in einer universitären Unfallchirurgie darzustellen.

**Methodik:**

Basierend auf den Entgelten je tariflicher Eingruppierung 2024 sowie modellhaften Annahmen der ärztlichen und pflegerischen Personalbesetzung einer universitären Unfallchirurgie wurden die Personalkosten für eine OP-Minute ermittelt. Eine beispielhafte Modellrechnung zur Kalkulation der OP-Personalkosten wurde für die Gammanagelosteosynthese bei proximaler Femurfraktur und die winkelstabile Plattenosteosynthese bei proximaler Humerusfraktur erstellt. Für die Intensivstation und Peripherstation erfolgten die Berechnungen der Personalkosten je Bett und 24-h-Tag.

**Ergebnisse:**

Die Personalkosten je OP-Minute betrugen 5,63 €. Die Modellrechnungen ergaben für die Gammanagelosteosynthese bei proximaler Femurfraktur Personalkosten von 300 € (Ø 53 OP-Minuten) und für die winkelstabile Plattenosteosynthese bei proximaler Humerusfraktur Personalkosten von 567 € (Ø 101 OP-Minuten). Die Personalkosten je 24-h-Tag auf der Intensivstation betrugen je Bett 807 €, auf der Peripherstation 192 €.

**Schlussfolgerung:**

Mit dem dargestellten Kalkulationsmodell ist eine zweckmäßige Berechnung der Personalkosten für die Bereiche OP und Station möglich. Unter Berücksichtigung der strukturellen und personellen Rahmenbedingungen ist das Modell auch auf Krankenhäuser anderer Versorgungsstufen übertragbar und kann als Grundlage für einen kosteneffizienten Personaleinsatz dienen.

**Graphic abstract:**

**Figure:**
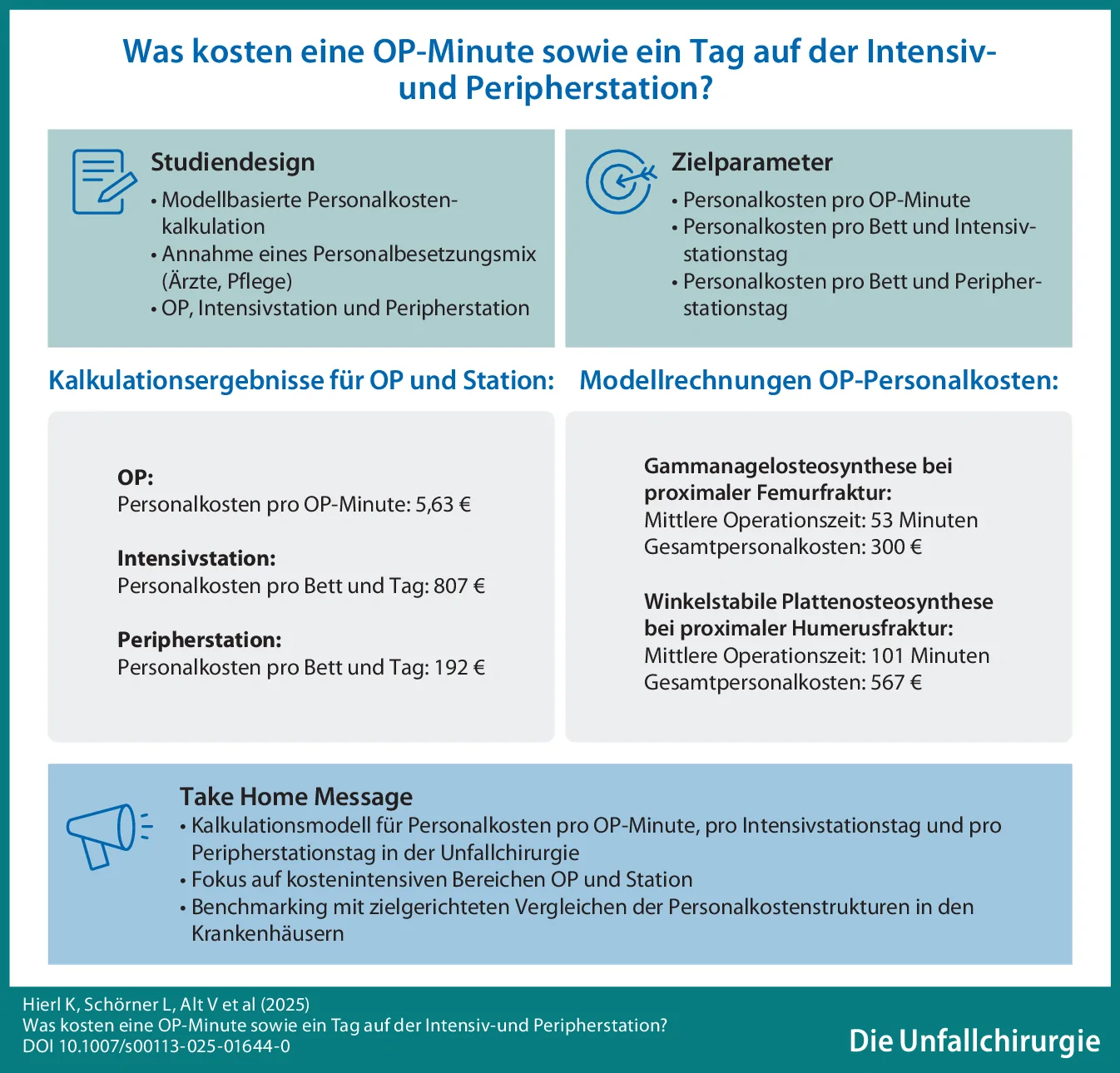

## Abstract (en)

**Background:**

The majority of the total costs in German hospitals are accounted for with around two thirds for personnel costs for medical and nursing services. The aim of this study was to present a health-economic model for physician and nursing service costs for the cost-intensive areas operating room (OR), intensive care unit and peripheral ward in a university orthopedic trauma department.

**Methods:**

Based on the remuneration per collective bargaining classification in 2024 and corresponding assumptions of the physician and nursing staffing for a university orthopedic trauma department, the staffing costs of 1min in the OR were determined. An exemplary model for the calculation of surgical staff costs was created for gamma nail osteosynthesis for proximal femoral fractures and angle-stable plate osteosynthesis for proximal humeral fractures. For the intensive care unit and peripheral ward, the staffing costs per bed were calculated for a 24‑h day.

**Results:**

The staffing costs per minute in the OR amounted to 5.63 €. The model calculations resulted in staffing costs of 300 € for gamma nail osteosynthesis for proximal femoral fractures (Ø 53 OR minutes) and staffing costs of 567 € for angle-stable plate fixation for proximal humeral fractures (Ø 101 OR minutes). The staffing costs for a 24‑h day amounted to 807 € per bed in the intensive care unit and 192 € for the peripheral ward.

**Conclusion:**

On the basis of the model presented, it is possible to calculate the staffing costs in the operating room as well as in the intensive care unit and peripheral ward. Taking the structural and staff basic conditions into account, the model can also be transferred to hospitals at other levels of care and can serve as a basis for a cost-effective deployment of staff.

In der Traumatologie stellen die Bereiche OP und Station die beiden größten Kostenblöcke bei der Behandlung dar, wobei die Personalkosten einen sehr hohen Anteil ausmachen. Vor dem Hintergrund der aktuellen wirtschaftlichen Lage mit steigendem ökonomischen Druck auf die deutschen Krankenhäuser ist ein kosteneffizienter Personaleinsatz im stationären Sektor von zunehmender Bedeutung. In diesem Beitrag wird ein Modell zur zweckmäßigen Personalkostenkalkulation von Ärztlichem Dienst und Pflegedienst vorgestellt.

## Einleitung

Die stationäre Krankenversorgung in Deutschland ist ein sehr personalintensiver Dienstleistungssektor, der etwa 1,2 Mio. Beschäftigte aufweist, mit einem Anstieg des Krankenhauspersonals um rund 16 % im Zeitraum von 2008 bis 2018 [13]. Laut Statistischem Bundesamt machten die Personalkosten in Höhe von 77,6 Mrd. € im Jahr 2021 mit knapp 62 % den größten Anteil der Gesamtkosten in den Krankenhäusern aus, wovon rund zwei Drittel der Personalkosten auf den Ärztlichen Dienst (31,4 %) und den Pflegedienst (32,5 %) entfielen [20].

Die PwC-Studie 2019 zum Krankenhaus-Benchmarking unterstreicht die zentrale Bedeutung der Personalkosten für die deutschen Krankenhäuser. Bei der Analyse der Finanzkennzahlen für die Jahre 2017/2018 fielen von 100 € Umsatzerlösen durchschnittlich etwa 60 € für Personalkosten an, woraus sich eine Personalaufwandsquote von knapp 60 % ergibt [16].

Um eine hohe Qualität der Krankenversorgung sicherzustellen und eine ökonomische Leistungserbringung in den Kliniken zu erreichen, sind eine zielorientierte Ausgestaltung der Prozesse und ein effizienter Personaleinsatz unabdingbar. Aufgrund der hohen Personalintensität in den Krankenhäusern sollte eine adäquate Personalbedarfsermittlung darauf abzielen, unter Sicherstellung einer bedarfsgerechten, effizienten und qualitativ hochwertigen medizinischen Behandlung den minimal erforderlichen Bedarf an Personal zu ermitteln [6].

Basierend auf quantitativen Kennzahlen und Indikatoren ist das operative Personal-Controlling im Hinblick auf die Planung und Steuerung der Personalkosten mit Orientierung am Tagesgeschäft in den Krankenhäusern für das Kosten- und Effizienz-Controlling verantwortlich [10, 12].

In der Traumatologie stellen die OP-Kosten und die Kosten für die stationäre Versorgung, hierbei v. a. die Personalkosten, die beiden größten Kostenblöcke bei der Behandlung dar [1, 4]. In einer Studie von Aigner et al. betrugen die Gesamtkosten pro Patient mit proximaler Femurfraktur 8853 €, wovon der Großteil mit 5828 € auf die Station und 1972 € auf den OP-Bereich entfielen [1]. Dorgham et al. analysierten die Krankenhausbehandlungskosten von proximalen Femurfrakturen und identifizierten als Hauptkomponenten die Personalkosten mit einem Anteil von 40 %, gefolgt von den Implantatkosten mit 25,9 % und den OP-Kosten mit 10 % [4].

Aufgrund der demografischen Entwicklung ist mit einem Anstieg an geriatrischen Frakturen, z. B. am proximalen Femur oder am proximalen Humerus, und demzufolge mit einer zunehmenden Zahl an pflegeintensiven und medizinisch aufwendigen Behandlungsfällen zu rechnen. Diese sind meist mit langen stationären Verweildauern, hohem Personaleinsatz von ärztlicher und pflegerischer Seite sowie mit hohen Behandlungskosten verbunden, sodass die Versorgung geriatrischer Frakturpatienten in der Regel nicht kostendeckend zu erbringen ist. Da geriatrische Patienten mit proximalen Femurfrakturen und proximalen Humerusfrakturen meist eine Vielzahl an behandlungsbedürftigen Komorbiditäten aufweisen, sind die Möglichkeiten zur Verweildauerreduktion und Kostensenkung insbesondere im Personalbereich für die Krankenhäuser limitiert [1].

Für eine kostenorientierte Personalsteuerung in der Unfallchirurgie sollten daher neben den OP-Kosten v. a. auch die Personalkosten auf der Intensivstation und der Peripherstation berücksichtigt werden. Zur Ermittlung der relevanten Kennzahlen OP-Minuten-Preis für unfallchirurgische Eingriffe sowie Kosten pro Tag auf einer Intensiv- und Peripherstation sind in der aktuellen Literatur kaum Daten vorhanden.

Pförringer et al. kalkulierten einen OP-Minuten-Preis von 12,48 € für ein überregionales universitäres Traumazentrum, der aus den OP-Gesamtkosten dividiert durch die gesamten OP-Minuten pro Jahr berechnet wurde [14]. Dabei machten die Personalkosten einen Anteil von mindestens 60 % an der Kostenkalkulation aus.

Neben den Personalkosten stellen die Implantate gerade in der Traumatologie einen großen Kostenfaktor im OP-Bereich dar, wie Shi et al. für die Versorgung von proximalen Humerusfrakturen mittels inverser Schulterprothese im Vergleich zur Plattenosteosynthese zeigen konnten [19].

An den Gesamtbehandlungskosten sind neben den OP-Kosten die Kosten für die stationäre Behandlung der Patienten der größte Punkt, wobei hier mit 38–48 % ein großer Anteil auf die Personalkosten für den Pflegedienst entfällt [11]. Insbesondere die pflegeintensiven und aufwendigen Behandlungen auf der Intensivstation verursachen hohe Kosten.

Bezüglich der Kosten pro Intensivstationstag finden sich in der Literatur sehr unterschiedliche Angaben. Bruyneel et al. analysierten die Pflegepersonalkosten und das Patienten-Outcome auf Intensivstationen in belgischen Krankenhäusern und kalkulierten Pflegekosten von durchschnittlich 748 € pro Bett und Tag für eine mittlere Anzahl von 16 Intensivbetten/Station [2]. Die Autoren kamen außerdem zu dem Ergebnis, dass höhere Kosten für speziell ausgebildete Pflegekräfte auf der Intensivstation mit signifikant geringeren Mortalitätsraten einhergehen.

Da die Pflege der Intensivpatienten sehr aufwendig und eine dementsprechend hohe Personalausstattung erforderlich ist, stellen die Personalkosten für den Pflegedienst mit rund 57 % den Hauptanteil der direkten Kosten bei der intensivstationären Behandlung dar [3, 9]. Als Faktoren, die zusätzlich mit höheren Behandlungskosten auf der Intensivstation assoziiert sind, wurden u. a. mechanische Beatmung, außerplanmäßige bzw. notfallmäßige Operationen sowie die Behandlung in Kliniken der maximalen Versorgungsstufe identifiziert [3, 9].

Im internationalen Vergleich von OECD-Ländern variieren die Gesamtkosten pro Tag auf der Intensivstation sehr stark zwischen 221 und 4322 €, da in den Ländern unterschiedliche Methoden zur Erfassung der Kostendaten und verschiedene Studiendesigns eingesetzt werden, um die Kosten für eine intensivstationäre Behandlung zu kalkulieren [8]. Zur Beurteilung der Kosteneffizienz hinsichtlich des Personaleinsatzes auf der Intensivstation sollten möglichst standardisierte und international gültige Methoden zur Kostenerfassung etabliert werden.

Zur Abschätzung der Pflegepersonalkosten auf der Peripherstation konnten Saville et al. mittels Computersimulation zeigen, dass Einrichtungen mit geringer Ausstattung an Pflegekräften und flexibler, bedarfsadaptierter Personalbesetzung zwar Personalkosten einsparen konnten, aber eine geringe Anzahl an festangestellten Pflegekräften und der flexible Rückgriff auf beispielsweise Zeitarbeitskräfte die Versorgungsqualität und die Patientensicherheit gefährden [18]. Trotz der Notwendigkeit eines kostenorientierten Personaleinsatzes angesichts des hohen ökonomischen Drucks auf die Krankenhäuser ist die Aufrechterhaltung einer hohen medizinischen Behandlungsqualität und der Patientensicherheit unumgänglich.

Vor diesem Hintergrund soll in der vorliegenden Arbeit ein Modell zur Kalkulation der Personalkosten von Ärztlichem Dienst und Pflegedienst für die Bereiche OP, Intensivstation und Peripherstation in einer universitären Unfallchirurgie dargestellt werden.

Es soll die Frage geklärt werden, wie viel eine OP-Minute sowie ein Tag auf der Intensiv- und Peripherstation kosten. Anhand einer Modellrechnung sollen die OP-Personalkosten beispielhaft für zwei unfallchirurgische operative Eingriffe am proximalen Femur und proximalen Humerus kalkuliert werden.

## Methodik

Für die Bereiche OP, Intensivstation und Peripherstation einer deutschen unfallchirurgischen Universitätsklinik wurden modellhafte Kalkulationen der Personalkosten für den Ärztlichen Dienst und Pflegedienst erstellt.

Dabei wurden grundsätzlich die Entgelte je Eingruppierung, die im Tarifvertrag des Jahres 2024 (TV‑L und TV-Ärzte Unikliniken) für die verschiedenen Berufsgruppen (Ärztlicher Dienst, Pflegedienst) geregelt sind, zugrunde gelegt. Zusätzlich dazu wurde ein Arbeitgeberanteil zur Sozialversicherung von jeweils 25,59 % angesetzt (Tab. 1, 2 und 3). Die Kalkulationen wurden mit dem Programm Excel Version 16 (Microsoft Corporation, Redmond, USA) angefertigt.

**Tab. 1:** Entgelte je Eingruppierung 2024 (in Euro) – Personal OP

Funktion	Oberarzt Unfallchirurgie	Facharzt Unfallchirurgie	Assistenzarzt Unfallchirurgie	Oberarzt Anästhesie	Facharzt Anästhesie	Assistenzarzt Anästhesie	Fachkrankenpflege OP	Fachkrankenpflege Anästhesie
Eingruppierung (*Beachte*: Annahme)	Ä 3/2	Ä 2/2	Ä 1/4	Ä 3/2	Ä 2/2	Ä 1/4	KR 9/5	KR 9/5
Grundvergütung	9202,19	7520,68	6137,18	9202,19	7520,68	6137,18	4017,47	4017,47
Zulage in Bayern	100,00	50,00	–	100,00	50,00	–	–	–
Uniklinikazulage	–	–	–	–	–	–	143,92	143,92
Zulage, OP	–	–	–	–	–	–	45,00	45,00
Erschwerniszulage	–	–	–	–	–	–	–	–
Gesamt/Monat	9302,19	7570,68	6137,18	9302,19	7570,68	6137,18	4206,39	4206,39
Faktor JSZ (%)	0,00	0,00	0,00	0,00	0,00	0,00	74,35	74,35
Jahressonderzahlung	–	–	–	–	–	–	3127,45	3127,45
Gesamt/Jahr, inkl. JSZ	111.626,28	90.848,16	73.646,16	111.626,28	90.848,16	73.646,16	53.604,13	53.604,13
SVAG-Anteil (%)	25,59	25,59	25,59	25,59	25,59	25,59	25,59	25,59
Gesamt/Jahr, inkl. JSZ + SVAG	140.191,45	114.096,20	92.492,21	140.191,45	114.096,20	92.492,21	67.321,43	67.321,43
Nettojahresarbeitszeit^1^ (h)	1680	1680	1680	1680	1680	1680	1540	1540
PK/h	83,45	67,91	55,05	83,45	67,91	55,05	43,72	43,72
PK/min	1,39	1,13	0,92	1,39	1,13	0,92	0,73	0,73

*JSZ* Jahressonderzahlung, *PK* Personalkosten, *SVAG* Sozialversicherung Arbeitgeberanteil

^1^ Mit Ausfallquote 20 %

**Tab. 2:** Entgelte je Eingruppierung 2024 (in Euro) – Personal Intensivstation

Funktion	Pflegefachkraft ohne FWB	Pflegefachkraft mit FWB	Pflegefachkraft mit/ohne FWB	Stationsleitung	Clinical Nurse Coordinator	Qualifizierter Transportdienst	Stationsassistenz (MFA)	Praxisanleiter	Krankenpflegeschüler	Studentische Hilfskraft	Oberarzt	Assistenzarzt
Eingruppierung (*Beachte*: Annahme)	KR 8/5	KR 9/5	KR 8/5 + 9/5^2^	KR 14/5	KR 8/5 + 9/5^2^	KR 8/5 + 9/5^2^	E 5/5	KR 8/5 + 9/5^2^	2. Lehrjahr	KR 5/3	Ä 3/2	Ä 1/4
Grundvergütung	3730,76	4017,47	3874,12	5431,01	3874,12	3874,12	3200,48	3874,12	1313,37	2751,08	9202,19	6137,18
Zulage in Bayern	–	–	–	–	–	–	–	–	–	–	100,00	–
Uniklinikazulage	143,92	143,92	143,92	143,92	143,92	143,92	–	143,92	–	–	–	–
Schichtzulage	–	–	–	–	–	–	–	–	–	–	–	–
Wechselschichtzulage	150,00	150,00	150,00	–	–	–	–	–	–	–	–	–
CM-Zulage	–	–	–	–	–	–	–	–	–	–	–	–
Gesamt/Monat	4024,68	4311,39	4168,04	5574,93	4018,04	4018,04	3200,48	4018,04	1313,37	2751,08	9302,19	6137,18
Faktor JSZ (%)	88,14	74,35	74,35	32,53	74,35	74,35	88,14	74,35	95,00	88,14	0,00	0,00
Jahressonderzahlung	3547,35	3205,52	3098,93	1813,52	2987,41	2987,41	2820,90	2987,41	1247,70	2424,80	–	–
Gesamt/Jahr, inkl. JSZ	51.843,51	54.942,20	53.115,35	68.712,68	51.203,83	51.203,83	41.226,66	51.203,83	17.008,14	35.437,76	111.626,28	73.646,16
SVAG-Anteil (%)	25,59	25,59	25,59	25,59	25,59	25,59	25,59	25,59	25,59	25,59	25,59	25,59
Gesamt/Jahr, inkl. JSZ + SVAG	65.110,27	69.001,91	66.707,57	86.296,26	64.306,89	64.306,89	51.776,57	64.306,89	21.360,52	44.506,29	140.191,45	92.492,21
Nettojahresarbeitszeit^1^ (h)	1540	1540	1540	1540	1540	1540	1540	1540	1540	1540	1680	1680
PK/h	42,28	44,81	43,32	56,04	41,76	41,76	33,62	41,76	13,87	28,90	83,45	55,05
PK/min	0,70	0,75	0,72	0,93	0,70	0,70	0,56	0,70	0,23	0,48	1,39	0,92

*CM* Case Management, *FWB* Fachweiterbildung, *JSZ* Jahressonderzahlung, *MFA* Medizinische Fachangestellte, *PK* Personalkosten, *SVAG* Sozialversicherung, Arbeitgeberanteil

^1^ Mit Ausfallquote 20 %

^2^ Annahme: Mittelwert

**Tab. 3:** Entgelte je Eingruppierung 2024 (in Euro) – Personal Peripherstation

Funktion	Pflegefachkraft	Pflegefachhelfer	Case-Management	Stationsassistenz (MFA)	Qualifizierter Transportdienst	Stationsleitung	Assistenzarzt
Eingruppierung (*Beachte*: Annahme)	KR 8/5	KR 6/3	KR 8/5	E 5/5	KR 7/4	KR 13/5	Ä 1/4
Grundvergütung	3730,76	2892,02	3730,76	3200,48	3498,17	5073,15	6137,18
Zulage in Bayern	–	–	–	–	–	–	–
Uniklinikazulage	143,92	143,92	143,92	–	143,92	143,92	–
Schichtzulage	–	–	–	–	–	60,00	–
Wechselschichtzulage	150,00	150,00	–	–	–	–	–
CM-Zulage	–	–	150,00	–	–	–	–
Gesamt/Monat	4024,68	3185,94	4024,68	3200,48	3642,09	5277,07	6137,18
Faktor JSZ (%)	88,14	88,14	88,14	88,14	88,14	32,53	0,00
Jahressonderzahlung	3547,35	2808,09	3547,35	2820,90	3210,14	1716,63	–
Gesamt/Jahr, inkl. JSZ	51.843,51	41.039,37	51.843,51	41.226,66	46.915,22	65.041,47	73.646,16
SVAG-Anteil (%)	25,59	25,59	25,59	25,59	25,59	25,59	25,59
Gesamt/Jahr, inkl. JSZ + SVAG	65.110,27	51.541,34	65.110,27	51.776,57	58.920,82	81.685,58	92.492,21
Nettojahresarbeitszeit^1^ (h)	1540	1540	1540	1540	1540	1540	1680
PK/h	42,28	33,47	42,28	33,62	38,26	53,04	55,05
PK/min	0,70	0,56	0,70	0,56	0,64	0,88	0,92

*CM* Case Management, *JSZ* Jahressonderzahlung, *MFA* Medizinische Fachangestellte, *PK* Personalkosten, *SVAG* Sozialversicherung, Arbeitgeberanteil

^1^ Mit Ausfallquote 20 %

Personalkosten, die außerhalb der medizinischen Versorgung anfallen, z. B. für Verwaltung oder Reinigung, wurden nicht mitkalkuliert. Daneben blieben weitere Kostenblöcke der Behandlung wie Kosten für Implantate, Arzneimittel oder Energie unberücksichtigt, da der Fokus in dieser Arbeit auf den Kosten des direkt an der medizinischen Behandlung beteiligten Personals lag.

### Personalkosten OP

Anhand der in Tab. 4 dargestellten Annahme der personellen Besetzung einer unfallchirurgischen Operation wurden die einzelnen Kosten je OP-Minute für das an dem Eingriff beteiligte ärztliche Personal (Facharzt Anästhesie, Oberarzt und Assistenzarzt Unfallchirurgie) und pflegerische Personal (Fachkrankenpflege OP und Anästhesie) sowie die gesamten Personalkosten je OP-Minute ermittelt.

**Tab. 4:** Personalbesetzung und Personalkosten (in Euro) für den Ärztlichen Dienst und Pflegedienst – OP

Personalbesetzung OP (*Beachte*: Annahme)				
Facharzt Anästhesie	Oberarzt Unfallchirurgie	Assistenzarzt Unfallchirurgie	Fachkrankenpflege OP	Fachkrankenpflege Anästhesie
1	1	1	2	1
*Personalkosten je OP-Minute*				
1,13	1,39	0,92	1,46	0,73
*Personalkosten gesamt je OP-Minute*				
5,63				

### Beispiele: Modellrechnungen OP-Personalkosten

Zur Veranschaulichung wurden für die zwei Beispiele Gammanagelosteosynthese bei proximaler Femurfraktur und winkelstabile Plattenosteosynthese bei proximaler Humerusfraktur unter Berücksichtigung der mittleren OP-Minuten modellhafte Berechnungen der OP-Personalkosten durchgeführt.

Zur Ermittlung der durchschnittlichen OP-Minuten der beiden operativen Eingriffe untersuchten wir zuvor im Rahmen einer Kosten-Erlös-Analyse 61 Fälle mit Gammanagelosteosynthese bei proximaler Femurfraktur sowie 32 Fälle mit winkelstabiler Plattenosteosynthese bei proximaler Humerusfraktur, die im Zeitraum vom Januar 2020 bis Dezember 2022 in dem betrachteten Krankenhaus operiert wurden und keine erlösrelevanten Komorbiditäten aufwiesen. Die Kalkulationen der Personalkosten basierten auf entsprechenden Annahmen der Personalbesetzung von ärztlicher und pflegerischer Seite; sie besitzen keine Allgemeingültigkeit (Tab. 5).

**Tab. 5:** Beispiele: Modellrechnung OP-Personalkosten (in Euro) für Gammanagelosteosynthese bei proximaler Femurfraktur und winkelstabile Plattenosteosynthese bei proximaler Humerusfraktur

Beispiele		Personalbesetzung je OP					Personalkosten je OP					
Operativer Eingriff	Mittlere OP-Minuten	FA, ANÄ	OA, UCH	AA, UCH	FKP, OP	FKP, ANÄ	FA, ANÄ	OA, UCH	AA, UCH	FKP, OP	FKP, ANÄ	Gesamt
Gammanagelosteosynthese	*53*	1	1	1	2	1	60,12	73,95	48,94	77,67	38,84	299,52
Winkelstabile Plattenosteosynthese	*101*	1	1	1^1^	2	1	113,71	139,88	92,58	146,92	73,46	566,55

*AA* Assistenzarzt, *ANÄ* Anästhesie, *FA* Facharzt, *FKP* Fachkrankenpflege, *OA* Oberarzt, *UCH* Unfallchirurgie

^1^ Annahme: zusätzlich PJ-Student als Assistenz

### Personalkosten Intensivstation und Peripherstation

Die Personalkosten für eine Intensivstation sowie eine unfallchirurgische Peripherstation mit jeweils 24 Betten wurden kalkuliert. Die Berechnungen basierten für beide Stationsarten auf einem Dreischichtenmodell für den Pflegedienst. Für den Ärztlichen Dienst wurde auf der Intensivstation ein Zweischichtenmodell, auf der Peripherstation ein Einschichtmodell angesetzt. Der jeweiligen Personalbesetzung lagen entsprechende Annahmen einer Universitätsklinik, die keine Allgemeingültigkeit besitzen, zugrunde. Hierbei wurden die Vorgaben der Verordnung zur Festlegung von Pflegepersonaluntergrenzen in pflegesensitiven Bereichen in Krankenhäusern [17] eingehalten. Gemäß der Pflegepersonaluntergrenzen-Verordnung (PpUGV) wurde für die unfallchirurgische Peripherstation schichtbezogen ein Verhältnis von Patienten zu einer Pflegekraft von 10 zu 1 (Tagschicht) und 20 zu 1 (Nachtschicht) angesetzt. Für die Intensivmedizin wurde für die Tagschicht ein Verhältnis von 2 zu 1 und für die Nachtschicht von 3 zu 1 berücksichtigt.

Folgende Annahmen wurden für die Personalkostenkalkulation der Intensivstation getroffen (Tab. 6):

- gleiche Besetzungsstärke von Pflegefachkräften werktags unter der Woche und am Wochenende/Feiertag (365 Tage pro Jahr),
- pflegerische Leitung, Clinical Nurse Coordinator, qualifizierter Transportdienst, Stationsassistenz, Praxisanleiter, Krankenpflegeschüler und studentische Hilfskräfte nur werktags unter der Woche (250 Tage/Jahr) im Einsatz,
- gleiche Besetzungsstärke von Oberärzten werktags unter der Woche und am Wochenende/Feiertag (365 Tage/Jahr),
- gleiche Besetzungsstärke von Assistenzärzten im Nachtdienst werktags unter der Woche und am Wochenende/Feiertag (365 Tage/Jahr).

**Tab. 6:** Personalbesetzung Pflegedienst und Ärztlicher Dienst – Intensivstation

*Personalbesetzung – Pflegedienst: Dreischichtenmodell*	*Früh*	*Spät*	*Nacht*	*Stunden/Platz und Schicht*	*Stunde/Tag insgesamt*	*Tage/Jahr*	*Stunden/Jahr insgesamt*	*VK*
Pflegefachkraft (3-jährig examiniert mit/ohne FWB)	12	12	9	8	264	365	96.360	62,57
Stationsleitung (3-jährig examiniert mit FWB)	2	0	0	8	16	250	4000	2,60
Clinical Nurse Coordinator (3-jährig examiniert mit/ohne FWB)	1	0	0	8	8	250	2000	1,30
Qualifizierter Transportdienst (3-jährig examiniert mit FWB)	1	0	0	8	8	250	2000	1,30
Stationsassistenz (MFA)	3	0	0	8	24	250	6000	3,90
Praxisanleiter (3-jährig examiniert mit/ohne FWB)	1	0	0	8	8	250	2000	1,30
Krankenpflegeschüler	1	0	0	8	8	250	2000	1,30
Studentische Hilfskraft	2	0	0	8	16	250	4000	2,60
GESAMT							118.360	76,86
*Personalbesetzung – Ärztlicher Dienst: Zweischichtenmodell*	*Tag*		*Nacht*	*Stunden/Platz und Schicht*	*Stunden/Tag insgesamt*	*Tage/Jahr*	*Stunden/Jahr insgesamt*	*VK*
Oberarzt (Montag-Sonntag/Feiertag)	2		0	12	24	365	8760	5,21
Assistenzarzt (Montag-Freitag)	4		2	12	72	250	18.000	10,71
Assistenzarzt (Samstag-Sonntag/Feiertag)	2		2	12	48	115	5520	3,29
GESAMT							32.280	19,21

*FWB* Fachweiterbildung, *MFA* Medizinische Fachangestellte, *VK* Vollkraft

Der Ermittlung der Personalkosten für die Peripherstation lagen die folgenden Annahmen zugrunde (Tab. 7):

- gleiche Besetzungsstärke von Pflegefachkräften werktags unter der Woche und am Wochenende (365 Tage/Jahr),
- unterschiedliche Besetzungsstärke von Pflegefachhelfern werktags unter der Woche (250 Tage) und am Wochenende/Feiertag (115 Tage/Jahr),
- Casemanagement, Stationsassistenz, qualifizierter Transportdienst und Stationsleitung nur werktags unter der Woche (250 Tage/Jahr) im Einsatz.

**Tab. 7:** Personalbesetzung Pflegedienst und Ärztlicher Dienst – Peripherstation

*Personalbesetzung – Pflegedienst: Dreischichtenmodell*	*Früh*	*Spät*	*Nacht*	*Stunden/Platz und Schicht*	*Stunden/Tag insgesamt*	*Tage/Jahr*	*Stunden/Jahr insgesamt*	*VK*
Pflegefachkraft (3-jährig examiniert)	3	3	1,5	8	60	365	21.900	14,22
Case Management (3-jährig examiniert)	1	0	0	8	8	250	2000	1,30
Stationsassistenz (MFA)	1	0	0	8	8	250	2000	1,30
Qualifizierter Transportdienst (3-jährig examiniert)	1	0	0	8	8	250	2000	1,30
Stationsleitung (3-jährig examiniert)	1	0	0	8	8	250	2000	1,30
Pflegefachhelfer (einjährig examiniert, Montag bis Freitag)	2	1	0	8	24	250	6000	3,90
Pflegefachhelfer (einjährig examiniert, Samstag, Sonntag und Feiertag)	1	1	0	8	16	115	1840	1,19
GESAMT							35.900	23,31
*Personalbesetzung – Ärztlicher Dienst: Einschichtmodell*	*Tag*		*Nacht*	*Stunden/Platz und Schicht*	*Stunden/Tag insgesamt*	*Tage/Jahr*	*Stunden/Jahr insgesamt*	*VK*
Assistenzarzt (Montag-Freitag)	1		0	8	8	250	2000	1,19
Assistenzarzt (Samstag-Sonntag/Feiertag)	1		0	3	3	115	345	0,21
GESAMT							2345	1,40

*MFA* Medizinische Fachangestellte, *VK* Vollkraft

Für beide Stationsarten wurden die Personalkosten für den Pflegedienst und den Ärztlichen Dienst für einen 24-h-Tag, gemittelt auf eine 7‑Tage-Woche, mit einer unterschiedlichen personellen Besetzung von Montag bis Freitag sowie Samstag, Sonntag und Feiertag berechnet.

Durch Division mit der Bettenanzahl (24/Station) wurden die anteiligen Personalkosten je Bett für einen 24-h-Tag auf der Intensivstation und der Peripherstation kalkuliert.

## Ergebnisse

### Personalkosten OP

Die kalkulierten gesamten Personalkosten je OP-Minute betrugen 5,63 € und setzten sich aus den jeweiligen Minutenkosten des beteiligten Ärztlichen Dienstes (Facharzt Anästhesie: 1,13 €, Oberarzt Unfallchirurgie: 1,39 €, Assistenzarzt Unfallchirurgie: 0,92 €) und Pflegedienstes (Fachkrankenpflege OP: 1,46 €, Fachkrankenpflege Anästhesie: 0,73 €) zusammen (Tab. 4).

### Beispiele: Modellrechnungen OP-Personalkosten

Für die Gammanagelosteosynthese bei proximaler Femurfraktur wurden bei einer mittleren OP-Dauer von 53 min Personalkosten von 300 € kalkuliert. Die Personalkosten für die winkelstabile Plattenosteosynthese bei proximaler Humerusfraktur lagen bei 567 € bei einer durchschnittlichen OP-Dauer von 101 min (Tab. 5).

Die Operationszeiten beziehen sich auf die Schnitt-Naht-Zeit der Eingriffe. Bei der Berechnung der durchschnittlichen Operationszeiten blieben die Zeiten für die anästhesiologische Vor- oder Nachbereitung im Rahmen der Ein- und Ausleitung unberücksichtigt.

### Personalkosten Intensivstation

Für den Pflegedienst auf der Intensivstation wurden Personalkosten von 14.715 € für einen 24-h-Tag (Montag bis Freitag) bzw. von 11.436 € (Samstag, Sonntag und Feiertag) berechnet. Gemittelt auf eine 7‑Tage-Woche lagen die Pflegepersonalkosten bei 13.778 € pro 24-h-Tag (Tab. 8).

**Tab. 8:** Personalkosten (in Euro) Pflegedienst und Ärztlicher Dienst – Intensivstation

	Personalkosten (PK)	PK für einen Tag (24 h) Mo–Fr	PK für einen Tag (24 h) Sa–So/FT	PK für einen Tag (24 h) Mo–So/FT
*Personalbesetzung – Pflegedienst: Dreischichtenmodell*				
Pflegefachkraft (3-jährig examiniert mit/ohne FWB)	4.173.988,15	11.435,58	11.435,58	11.435,58
Stationsleitung (3-jährig examiniert mit FWB)	179.225,73	896,58	–	640,42
Clinical Nurse Coordinator (3-jährig examiniert mit/ohne FWB)	83.515,44	334,06	–	238,62
Qualifizierter Transportdienst (3-jährig examiniert mit FWB)	83.515,44	334,06	–	238,62
Stationsassistenz (MFA)	201.726,88	806,91	–	576,36
Praxisanleiter (3-jährig examiniert mit/ohne FWB)	83.515,44	334,06	–	238,62
Krankenpflegeschüler	27.740,94	110,96	–	79,26
Studentische Hilfskraft	115.600,74	462,40	–	330,29
GESAMT	4.948.828,76	14.714,63	11.435,58	13.777,76
*Personalbesetzung – Ärztlicher Dienst: Zweischichtenmodell*				
Oberarzt (Montag bis Sonntag/Feiertag)	730.998,25	2002,73	2002,73	2002,73
Assistenzarzt (Montag bis Freitag)	990.987,99	3963,95	–	2831,39
Assistenzarzt (Samstag, Sonntag und Feiertag)	303.902,98	–	2642,63	755,04
GESAMT	2.025.889,22	5966,69	4645,37	5589,17
*Personalbesetzung: Pflegedienst* *+* *Ärztlicher Dienst*				
GESAMT	6.974.717,98	20.681,32	16.080,95	19.366,93
**PK (PD) pro Bett und Tag (24** **h) Mo–So/FT**	**574,07**			
**PK (ÄD) pro Bett und Tag (24** **h) Mo–So/FT**	**232,88**			
**PK (PD** **+** **ÄD) pro Bett und Tag (** **h) Mo–So/FT**	**806,96**			

*ÄD* Ärztlicher Dienst, *Fr* Freitag, *FT* Feiertag, *FWB* Fachweiterbildung, *MFA* Medizinische Fachangestellte, *Mo* Montag, *PD* Pflegedienst, *PK* Personalkosten, *Sa* Samstag, *So* Sonntag

Für den Ärztlichen Dienst auf der Intensivstation betrugen die Personalkosten von Montag bis Freitag 5967 € für einen 24-h-Tag und 4645 € von Samstag bis Sonntag/Feiertag. Bei Betrachtung einer 7‑Tage-Woche mit einer unterschiedlichen Besetzung werktags und am Wochenende/Feiertag wurden 5589 € je 24-h-Tag kalkuliert (Tab. 8).

Es ergaben sich Gesamtpersonalkosten von 19.367 € pro Tag (24 h) für den Pflegedienst und Ärztlichen Dienst für die gesamte Intensivstation sowie Personalkosten von 807 € je Bett pro 24-h-Tag auf der Intensivstation (Tab. 8).

### Personalkosten Peripherstation

Die Personalkosten für den Pflegedienst auf der Peripherstation lagen bei 4710 € pro 24-h-Tag (Montag bis Freitag) bzw. bei 3072 € (Samstag bis Sonntag/Feiertag). Es wurden gemittelte Personalkosten von 4242 € pro Tag (24 h) bei Betrachtung einer 7‑Tage-Woche mit einer unterschiedlichen Besetzung werktags und am Wochenende/Feiertag berechnet (Tab. 9).

**Tab. 9:** Personalkosten (in Euro) Pflegedienst und Ärztlicher Dienst – Peripherstation

	Personalkosten (PK)	PK für einen Tag (24 h) Mo–Fr	PK für einen Tag (24 h) Sa–So/FT	PK für einen Tag (24 h) Mo–So/FT
*Personalbesetzung – Pflegedienst: Dreischichtenmodell*				
Pflegefachkraft (3-jährig examiniert)	925.918,75	2536,76	2536,76	2536,76
Case Management (3-jährig examiniert)	84.558,79	338,24	–	241,60
Stationsassistenz (MFA)	67.242,29	268,97	–	192,12
Qualifizierter Transportdienst (3-jährig examiniert)	76.520,55	338,24	–	241,60
Stationsleitung (3-jährig examiniert)	106.085,17	424,34	–	303,10
Pflegefachhelfer (einjährig examiniert, Montag bis Freitag)	200.810,42	803,24	–	573,74
Pflegefachhelfer (einjährig examiniert, Samstag, Sonntag und Feiertag)	61.581,86	–	535,49	153,00
GESAMT	1.461.135,97	4709,79	3072,26	4241,92
*Personalbesetzung – Ärztlicher Dienst: Zweischichtenmodell*				
Assistenzarzt (Montag bis Freitag)	110.109,78	440,44	–	314,60
Assistenzarzt (Samstag, Sonntag und Feiertag)	18.993,94	–	165,16	47,19
GESAMT	129.103,71	440,44	165,16	361,79
*Personalbesetzung: Pflegedienst* *+* *Ärztlicher Dienst*				
GESAMT	1.590.239,68	5150,23	3237,42	4603,71
**PK (PD) pro Bett und Tag (24** **h) Mo–So/FT**	**176,75**			
**PK (ÄD) pro Bett und Tag (24** **h) Mo–So/FT**	**15,07**			
**PK (PD** **+** **ÄD) pro Bett und Tag (24** **h) Mo–So/FT**	**191,82**			

*ÄD* Ärztlicher Dienst, *Fr* Freitag, *FT* Feiertag, *MFA* Medizinische Fachangestellte, *Mo* Montag, *PD* Pflegedienst, *PK* Personalkosten, *Sa* Samstag, *So* Sonntag

Die Personalkosten für den Ärztlichen Dienst auf der Peripherstation betrugen von Montag bis Freitag 440 € je 24-h-Tag sowie 165 € von Samstag bis Sonntag/Feiertag. Gemittelt auf eine 7‑Tage-Woche ergaben sich Personalkosten von 362 € pro Tag (Tab. 9).

Für den Pflegedienst und Ärztlichen Dienst zusammen wurden Personalkosten von 4604 € je 24-h-Tag für die Station insgesamt sowie Kosten von 192 € je Bett pro Tag (24 h) auf der peripheren Station ermittelt (Tab. 9).

## Diskussion

In diesem Beitrag sollte eine modellhafte Berechnung der Personalkosten für die Bereiche OP, Intensivstation und Peripherstation in einer universitären Unfallchirurgie aufgezeigt werden, da die Personalkosten die wichtigste Kostenart in den Krankenhäusern darstellen und bei vielen betriebswirtschaftlichen Kalkulationen im stationären Sektor eine Rolle spielen.

Zunächst ist anzumerken, dass die Aussagekraft der vorliegenden Studie limitiert ist, da unsere Ergebnisse aufgrund des Modellcharakters der Personalkostenkalkulationen mit rein beispielhafter Besetzung des ärztlichen und pflegerischen Personals im OP und auf den Stationen keine universelle Gültigkeit besitzen, sondern lediglich als Anhaltspunkt für eine zweckmäßige Ermittlung der Personalkosten in den Krankenhäusern dienen können.

Grundsätzlich werden die Personalkosten in der Klinik neben der beruflichen Qualifikation des eingesetzten pflegerischen und ärztlichen Personals auch von ggf. anfallenden Zuschlägen, z. B. für Bereitschaftsdienste, Wochenend- oder Nachtarbeit, beeinflusst und können je nach zugrunde liegender Konstellation insbesondere nach oben variieren. Dies blieb bei den Kostenkalkulationen in dieser Studie unberücksichtigt, sodass unsere Ergebnisse den klinischen Alltag nicht umfassend widerspiegeln.

### Personalkosten OP

Unsere Berechnungen ergaben Personalkosten von 5,63 € je OP-Minute für eine unfallchirurgische Operation unter entsprechender Annahme der personellen Besetzung, die keine Allgemeingültigkeit besitzt. Durch Multiplikation der Minutenkosten mit der OP-Dauer lassen sich die Kosten für bestimmte Operationen mit der jeweiligen Personalbesetzung berechnen, was beispielsweise für zielgerichtete Vergleiche der OP-Personalkosten mit anderen Krankenhäusern genutzt werden kann.

Der OP-Minuten-Preis unserer Studie bezieht sich im Gegensatz zu der Arbeit von Pförringer et al. [14] ausschließlich auf die Kosten des direkt an dem Eingriff beteiligten ärztlichen und pflegerischen Personals ohne Einschluss der Implantatkosten sowie sonstiger Kosten, z. B. für Arzneimittel, und ist daher mit 5,63 € im Vergleich zu den angegebenen 12,48 € deutlich niedriger.

Da das Untersuchungsziel dieses Beitrags die Kalkulation der OP-Personalkosten unter Annahme eines definierten Personalbesetzungsmix war und nicht die Berechnung der OP-Kosten insgesamt, wurden die Kosten für Implantate und Arzneimittel sowie Sachkosten ausgeschlossen. Im Gegensatz zu den OP-Kostenanalysen von Pförringer et al. [14] und Shi et al. [19] lag der Fokus in der vorliegenden Arbeit auf den Personalkosten im OP-Bereich, da hierzu kaum wissenschaftliche Untersuchungen vorhanden sind.

Im Rahmen der beispielhaften Modellrechnungen wurden für die Gammanagelosteosynthese bei proximaler Femurfraktur Personalkosten von 300 € bei einer mittleren Operationsdauer von 53 min ermittelt. Aufgrund der längeren Operationszeit von durchschnittlich 101 min mit gleicher Personalbesetzung bei der winkelstabilen Plattenosteosynthese am proximalen Humerus waren die Personalkosten mit 567 € deutlich höher.

Zur Einordnung dieser Ergebnisse in die aktuelle DRG-Systematik dient ein Vergleich mit der Kalkulationsmatrix für Personalkosten im G‑DRG-Kosten-Tool 2024 [5]. Bei der Gammanagelosteosynthese betragen die kalkulierten Personalkosten in der entsprechenden DRG I08F im OP-Bereich 360 € für den Ärztlichen Dienst und 355 € für den Medizinisch-Technischen Dienst. In der DRG I13E (Winkelstabile Plattenosteosynthese bei proximaler Humerusfraktur) werden im Kosten-Tool OP-Personalkosten von 491 € (Ärztlicher Dienst) und 458 € (Medizinisch-Technischer Dienst) kalkuliert. Die modellhaft berechneten Personalkosten der vorliegenden Arbeit beziehen sich jedoch konkret auf die beispielhaft angegebene Personalbesetzung je OP (Ärztlicher Dienst und Pflegedienst) mit den jeweiligen Operaionszeiten. Durch die unterschiedlichen Kalkulationsansätze sind die Personalkosten für den OP-Bereich des DRG-Tools mit den Ergebnissen dieses Beitrags daher nicht direkt vergleichbar.

Im Allgemeinen hängen die OP-Gesamtkosten nicht nur von den OP-Minuten-Personalkosten, sondern natürlich auch von der Operationsdauer ab; diese kann aus verschiedenen Gründen, z. B. im Rahmen von Weiterbildungseingriffen, variieren [14, 15].

Neben personalbedingten und frakturmorphologischen Aspekten können auch eingriffsspezifische sowie patientenbezogene Faktoren zu Verlängerungen der Operationszeiten, die sich in operativen Mehrkosten für die Kliniken widerspiegeln, führen. Eine Studie von Gutwerk et al. zeigte, dass es bei der Versorgung proximaler Femurfrakturen bei steigendem Body-Mass-Index der Patienten zu einer linearen Verlängerung der Operationszeit sowie der stationären Verweildauer und somit zu einem kontinuierlichen Kostenanstieg kam, was im aktuellen aG-DRG-System nicht in ausreichendem Maß kompensiert wird [7].

### Personalkosten Intensivstation und Peripherstation

In unserer Studie lagen die Personalkosten je Bett auf der Intensivstation pro 24-h-Tag, gemittelt auf eine 7‑Tage-Woche, bei 807 € vs. 192 € je Bett pro Tag auf der Peripherstation.

Übertragen auf die Personalkosten in der Kalkulationsmatrix 2024 für die beispielhaft angesetzten DRG I08F und I13E zeigt sich beim Vergleich mit unseren Ergebnissen für den Bereich Normal- und Intensivstation aufgrund der unterschiedlichen Rechenansätze eine deutliche Differenz beim Ärztlichen Dienst (I08F: 732 € Normalstation, 76 € Intensivstation. I13E: 503 € Normalstation, 17 € Intensivstation). In unserer Studie betragen die Personalkosten für den Ärztlichen Dienst je Bett auf der Normalstation 15 € pro Tag sowie 233 € auf der Intensivstation. Bezogen auf die mittlere Verweildauer der angenommenen DRG I08F (9,2 Tage) und I13E (5,7 Tage) ließen sich somit ärztliche Personalkosten von 139 € bzw. 86 € pro Patient auf der Normalstation berechnen, ohne Berücksichtigung eines intensivstationären Aufenthalts. Während die Berechnungen im DRG-Tool pauschal die Kosten für den gesamten Ärztlichen Dienst für die Kostenstellenbereiche Normal- und Intensivstation, bezogen auf die mittlere Verweildauer der DRG, angeben, beziehen sich unsere Ergebnisse auf die Personalkosten der konkret angenommenen ärztlichen Besetzung (Assistenzarzt, Oberarzt) auf der Peripher- und Intensivstation.

Durch die Ausgliederung der Pflegepersonalkosten im aG-DRG-System ist ein direkter Vergleich der pflegerischen Personalkosten unserer Ergebnisse mit der Kalkulationsmatrix an dieser Stelle nicht möglich.

In Abhängigkeit von der Stationsgröße sind die Kosten je Bett sicherlich variabel und können je nach Bettenanzahl nach oben oder unten abweichen. Unsere Kostenkalkulationen beziehen sich auf eine chirurgisch-anästhesiologische Intensivstation sowie eine unfallchirurgische Normalstation mit jeweils 24 Betten, sodass insbesondere für die eher groß angenommene Zahl an Intensivbetten ein relativ hoher Kostenbetrag von 807 € je Bett resultierte.

In unserer Arbeit wurden im Gegensatz zu der Studie von Bruyneel et al. [2] zusätzlich die Personalkosten für den Ärztlichen Dienst berücksichtigt und die Kalkulationen für eine 24-Betten-Intensivstation durchgeführt, sodass der Betrag mit 807 € vs. 748 € etwas höher lag.

In der eingangs angeführten Arbeit von Saville et al. zur Analyse der Pflegepersonalkosten auf der Peripherstation in englischen Krankenhäusern lagen die Personalkosten für die Pflegekräfte im Durchschnitt bei 120–142 £ (entspricht 139–165 €) pro Patiententag in Abhängigkeit von der jeweiligen personellen Besetzung und den Personalstufen [18]. Dies lässt sich durchaus mit unseren Ergebnissen vergleichen. Abzüglich der ärztlichen Personalkosten von 15 € kalkulierten wir Pflegepersonalkosten von durchschnittlich 177 € pro Bett und Tag für den angenommenen Personalbesetzungsmix auf der Peripherstation.

## Limitationen

Diese Studie unterliegt einigen Limitationen. Die Kalkulationen der Personalkosten wurden modellhaft auf Basis der dargestellten Annahmen für die personelle Besetzung der operativen Eingriffe sowie auf der Intensivstation und Peripherstation für eine universitäre Unfallchirurgie durchgeführt und besitzen keine Allgemeingültigkeit. Die Personalbesetzung im Ärztlichen Dienst und im Pflegedienst gestaltet sich in der Realität in den Krankenhäusern aufgrund der unterschiedlichen Rahmenbedingungen sehr variabel, mit direkter Auswirkung auf die Personalkosten.

Die Personalkostenkalkulationen wurden ausschließlich für die Bereiche OP und Station erstellt, wobei diese die beiden größten Kostenblöcke bei unfallchirurgischen Behandlungen abbilden.

Indirekte Personalkosten, z. B. in Kostenstellen wie Reinigung und Verwaltung, wurden bei den Kalkulationen ausgeschlossen, wobei immanent unterstellt wurde, dass ein höherer oder niedrigerer Personaleinsatz in diesen Bereichen keinen Einfluss auf die Personalkosten des direkt an der medizinischen Behandlung im OP und auf der Station beteiligten Personals hat.

Einschränkend ist weiterhin zu sagen, dass die Modellrechnungen speziell für die Gammanagelosteosynthese bei proximaler Femurfraktur und die winkelstabile Plattenosteosynthese bei proximaler Humerusfraktur mit einem konstanten Personalbesetzungsmix durchgeführt wurden und andere operative Eingriffe mit variierender personeller Besetzung unberücksichtigt blieben.

## Fazit für die Praxis

In dem vorliegenden Beitrag wurde erstmals ein Kalkulationsmodell zur Berechnung der Personalkosten pro OP-Minute, pro Intensivstationstag und pro Peripherstationstag in einer unfallchirurgischen Universitätsklinik erstellt. Dabei lag der Fokus auf den Kosten für verschiedene Personalkategorien unter Annahme eines definierten Personalbesetzungsmix mit Herausrechnung sonstiger Kosten, beispielsweise für Implantate oder Arzneimittel.

Unter Berücksichtigung der strukturellen und personellen Rahmenbedingungen ist das Modell auf Krankenhäuser aller Versorgungsstufen übertragbar und kann im Rahmen von Benchmarking mit Vergleich und Analyse der Personalkostenstrukturen für die Bereiche OP sowie Station zur Kostenoptimierung und Effizienzsteigerung in den Kliniken verwendet werden.

## Data Availability

Die erhobenen Datensätze können auf begründete Anfrage in anonymisierter Form beim korrespondierenden Autor angefordert werden. Die Daten befinden sich auf einem Datenspeicher am Universitätsklinikum Regensburg.
